# Formation of Pt-Based Alloy Nanoparticles Assisted by Molybdenum Hexacarbonyl

**DOI:** 10.3390/nano11071825

**Published:** 2021-07-14

**Authors:** Gerard M. Leteba, David R. G. Mitchell, Pieter B. J. Levecque, Eric van Steen, Candace I. Lang

**Affiliations:** 1 Catalysis Institute, Department of Chemical Engineering, University of Cape Town, Cape Town 7700, South Africa; Pieter.levecque@uct.ac.za (P.B.J.L.); eric.vansteen@uct.ac.za (E.v.S.); 2 School of Engineering, Macquarie University, Sydney, NSW 2109, Australia; 3 Electron Microscopy Centre, Innovation Campus, University of Wollongong, Wollongong, NSW 2517, Australia; dmitchel@uow.edu.au

**Keywords:** alloys, nanoparticle, molybdenum hexacarbonyl, surfactants, STEM-EDXS, thermolytic synthesis

## Abstract

We report on an optimized, scalable solution-phase synthetic procedure for the fabrication of fine-tuned monodisperse nanostructures (Pt(NiCo), PtNi and PtCo). The influence of different solute metal precursors and surfactants on the morphological evolution of homogeneous alloy nanoparticles (NPs) has been investigated. Molybdenum hexacarbonyl (Mo(CO)_6_) was used as the reductant. We demonstrate that this solution-based strategy results in uniform-sized NPs, the morphology of which can be manipulated by appropriate selection of surfactants and solute metal precursors. Co-surfactants (oleylamine, OAm, and hexadecylamine, HDA) enabled the development of a variety of high-index faceted NP morphologies with varying degrees of curvatures while pure OAm selectively produced octahedral NP morphologies. This Mo(CO)_6_-based synthetic protocol offers new avenues for the fabrication of multi-structured alloy NPs as high-performance electrocatalysts.

## 1. Introduction

Recent research efforts in hydrogen polymer electrode membrane fuel cells (PEMFCs) have focused on the design of platinum (Pt)-based binary and ternary alloy nanoparticles (NPs) as catalysts. The aim is to develop NPs which are less costly than pure Pt NPs while exhibiting catalytic properties which are superior to commercially available Pt/C NP catalysts. A particular focus is acceleration of the sluggish oxygen reduction reaction (ORR) [1,2,3,4,5,6]. The most successful NPs reported to date, formed by the dilution of costly Pt with less-expensive 3d-transition metals, show good catalytic performance [2,7,8,9], but further improvement is sought. Optimising the functionality of alloy NP electrocatalysts, particularly for the ORR, requires an understanding of the solution-phase (wet-chemical) synthetic process [3,6,10,11]. Solution-phase synthesis of NPs for industrial applications should ideally result in NP structures with a high degree of crystallinity, narrow particle size distribution, uniform morphology and excellent dispersability in organic solvents [6,7,11,12,13,14]. However, the reduction of two or more different metal precursors in solution-based media often leads to the creation of core-shell, hetero-structured, or segregated NPs, rather than homogenous nanoalloys, due to differences in the standard reduction potentials of the metal precursors [12,15].

Synthesizing solid solution alloy NPs requires manipulation of the reaction kinetics (which influences the degree of nucleation and the subsequent growth mechanisms), which in turn dictates the morphology of the resulting NPs [10,16,17]. Examples include the co-reduction of two or more different metal precursor salts at a similar rate, achieved via the use of high temperatures (≥150 °C), and regulation of the nucleation and growth of NPs, achieved by the appropriate selection of surfactant ligands [8,9,16,18,19,20]. Recent research has shown that size- and morphology-controlled PtM (M = Ni, Co, Fe) NPs can be prepared by using tungsten carbonyl (W(CO)_6_) as the reductant in the presence of co-surfactants oleylamine and oleic acid [9,21,22]. The decomposition of W(CO)_6_ to W^0^, liberating gaseous CO, rapidly reduces Pt^2+^ to Pt^0^ (atoms/seeds) and this leads to fast Pt-nucleation [9,23]. W^0^ does not alloy with Pt^0^ under the reaction conditions and is used as the reducing agent and remains as an ionic species (W^n+^ or W^6+^) in the bulk growth organic reaction mixture [9], permitting thermodynamically controlled PtM NP growth [21]. CO is known to adsorb preferentially onto specific Pt facets, with strong selective binding on {100} facets and weak adsorption on {111} facets; and has been reported to influence NP morphology [24,25]. The use of W(CO)_6_ to liberate CO, however, has been reported to result in mostly cubic and octahedral NPs [9,21,22].

Here, we report a facile, one-pot organic solution-phase synthetic route, for the formation of binary and ternary Pt-based alloy NPs. Synthesis is assisted by the decomposition of Mo(CO)_6_ as the reductant of the various metal salts. Our synthetic protocol yielded Pt(NiCo), PtNi, and PtCo alloy NPs, solution-grown under similar reduction reaction conditions using a homogeneous mixture of surfactants (oleylamine (OAm) and hexadecylamine (HDA)). All NPs consisted of compositionally disordered solid solutions; but the morphologies were sensitive to synthesis variables, solute metal precursors and synthetic approaches. Unlike W(CO)_6_ which creates predominantly cubic and octahedral NPs, we demonstrate here that the reductant Mo(CO)_6_ produces a variety of concave high-index faceted NP morphologies with various degrees of branching growth. Furthermore, the use of Mo(CO)_6_ allowed us to investigate the nucleation and growth pathways of the NPs during synthesis by manipulating these variables and to optimise the synthesis. The synthetic protocol described thus offers the preparation of well-defined concave Pt-alloy NPs with great potential for future use in fuel cell devices.

## 2. Materials and Methods

The solution-phase synthetic approach used the following metal precursors (all purchased from Sigma-Aldrich): platinum acetylacetonate (Pt(acac)_2_, 97%), nickel (II) acetate tetrahydrate (Ni(Ac)_2_, 98%), cobalt (II) acetate tetrahydrate (Co(Ac)_2_, ≥99%), nickel (II) acetylacetonate (Ni(acac)_2_, 95%), cobalt (II) acetylacetonate (Co(acac)_2_, 97%), and molybdenum hexacarbonyl (Mo(CO)_6_, 98%). Oleylamine (OAm, 70%) and hexadecylamine (HDA, 90%) were employed as surfactants and benzyl ether (BE, 98%) as a high-boiling point solvent. Solvents (Sigma-Aldrich), such as absolute ethanol, acetone, and chloroform, used for precipitating, cleaning, and re-dispersing the particles, were all of analytical grade. All the chemicals were used as received without any further purification.

## 3. Synthesis

(a) Mo(CO)_6_-assisted synthesis of Pt-based alloy nanoparticles.

(i) Synthesis of binary Pt-based nanoparticles using precursor salts Ni(Ac)_2_ and Co(Ac)_2_. In a typical synthesis: precursor salts (Pt(acac)_2_ (0.08 g, 0.2 mmol) + Ni(Ac)_2_ (0.048 g, 0.2 mmol)) or (Pt(acac)_2_ (0.08 g, 0.2 mmol) + Co(Ac)_2_ (0.048 g, 0.2 mmol)) were dissolved in surfactants OAm (20 mL) and HDA (4.4 g) using BE (25 mL) as the high boiling point solvent. The resulting metal salt-surfactant-solvent reaction mixture was heated to 150 °C for 5–10 min under vigorous magnetic stirring in a round bottom flask. Mo(CO)_6_ (0.1 g, 0.38 mmol) was added, with the result that the pale-yellow homogeneous solution turned dark purple, and a smokey vapour was emitted, suggesting the evolution of gaseous CO. The bulk organic synthesis mixture was then heated to 240 °C at a heating rate of 10 °C/min, turning dark brown during the heating process. The resultant colloidal mixture was held at 240 °C for 60 min, with aliquot extraction from the bulk synthesis mixture every 15 min to investigate the growth mechanism of NPs. Thereafter, the colloidal medium was removed from the heat source and quenched using ice to limit any structural transformations occurring during the cooling process. Subsequently, the as-synthesized NPs were extracted from the synthesis media through flocculation by adding excess absolute ethanol and chloroform. After settling (typically 1–2 days), the excess organic solvents were decanted, and the particles were further cleaned by re-suspending in absolute ethanol. This colloidal refining process was performed 3 times. The black product was finally re-dispersed in chloroform, yielding a dark brown colloidal suspension.

Under the same synthesis conditions, ternary Pt(NiCo) NPs were synthesized by dissolving (Pt(acac)_2_ (0.08 g, 0.2 mmol) + Ni(Ac)_2_ (0.024 g, 0.1 mmol) + Co(Ac)_2_ (0.024 g, 0.1 mmol)) in OAm (20 mL) + HDA (4.4 g) + BE (25 mL). The simultaneous reduction process is similar to that described above for the preparation of binary alloy NPs.

(ii) Synthesis of binary and ternary Pt-based nanoparticles. This co-reduction approach in (a) (ii), (iii), (iv) is similar to the synthetic method described above in (a) (i), except for use of doubled HDA concentration. (iii) Synthesis of binary and ternary Pt-based nanoparticles. This synthetic approach for the formation of binary and ternary Pt-based nanoparticles is similar to that described in (a) (i) except that the precursor salts Ni(acac)_2_ and Co(acac)_2_ were used instead of Ni(Ac)_2_ and Co(Ac)_2_. (iv) Synthesis of binary and ternary Pt-based nanoparticles. The co-reduction process is similar to those described above in (a) (i) and (ii) using a single surfactant, OAm, without the use of HDA. All the purification procedures were similar to that described above in (i). (b) Thermal decomposition of organometallic precursors. This co-thermal decomposition technique (b) (i), (ii), (iii) and (iv) is similar to syntheses described above in (a) (i), (ii), (iii) and (iv), without the use of reductant Mo(CO)_6_ (see details in Supporting Information methods).

Nanostructural characterization techniques. The black powders of the as-synthesised and unsupported nanoparticles were deposited onto a silicon (Si) wafer support for powder X-ray diffraction (PXRD) patterns, recorded on a Bruker AXS D8 using Co K_α_ radiation (λ = 0.179026 nm). Specimens for scanning transmission electron microscopy (STEM) investigations were prepared by one-drop casting of a colloidal solution onto carbon-support films on copper grids. These were air dried under ambient conditions. Specimens were analysed using TEM, HRTEM and STEM on a JEOL ARM200F probe-corrected instrument, operating at 200 kV. The chemical compositions of individual nanoparticles were determined using energy dispersive X-ray spectroscopy (EDXS) in STEM mode. Spectrum imaging was used in which an EDS spectrum is obtained at each pixel in the STEM Image, to produce a 3D dataset. Rapid acquisition was used (5 s per frame) integrated over at least 100 frames. Image drift correction was applied after each frame. Scanning TEM (STEM) imaging used bright field and high angle annular dark field (HAADF) imaging modes. The bulk elemental compositions of the as-prepared NPs were investigated using a Varian 730-ES inductively coupled plasma-optical emission spectrometer (ICP-OES).

## 4. Results and Discussion

We compared the influence of molybdenum hexacarbonyl (Mo(CO)_6_, reductant) and thermolytic approaches on the morphological selectivity and size evolution of Pt-based alloy NPs. We also studied the effect of varying several synthesis parameters (time, amine surfactants (OAm and HDA), solute precursor salts (acetate and acetylacetonate)) while maintaining other parameters constant (solvent (BE), Pt salt (Pt(acac)_2_) and reduction temperature (240 °C)). In these studies, our efforts focused on manipulating the reduction kinetics of Pt and M (M = Ni and Co) precursors in order to circumvent uneven Ostwald ripening, or spontaneous nucleation and successive growth of segregated Pt and solute NPs. We used amine-based surfactants (OAm and HDA), which possess the same functional groups, for simultaneous control of composition, size distribution, monodispersity and faceting of the alloy NPs. A high boiling point solvent, benzyl ether (BE, boiling point: 298 °C), provided a homogeneous liquid phase for nucleation, growth, and controlled-interdiffusion of distinct metal atoms, hence providing a medium for homogeneous crystal growth [26].

## 5. Synthesis Using Mo(CO)_6_

**Figure 1:**
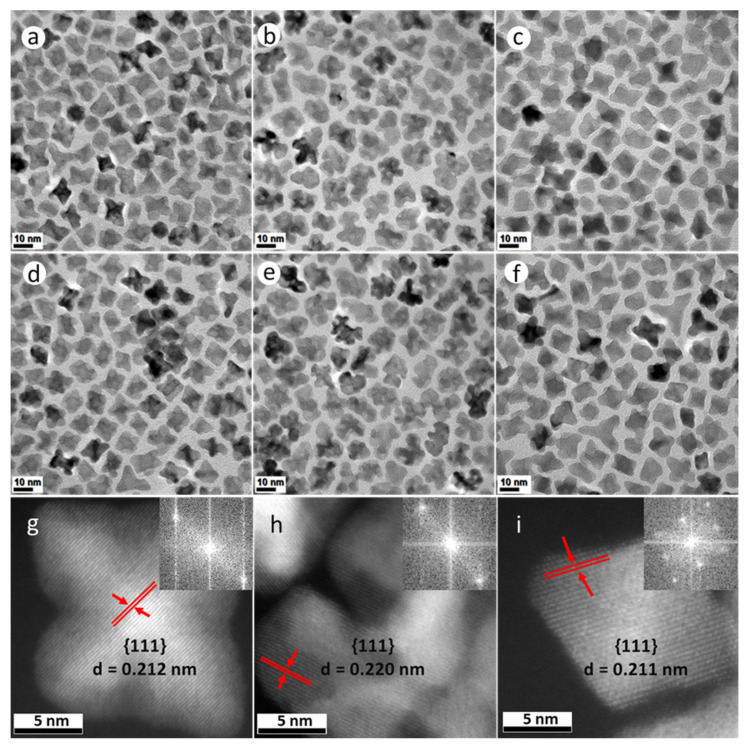
TEM bright field (BF) images of (**a**–**f**) PtNi, PtCo and Pt(NiCo) alloy NPs show insignificant morphological and size changes after the first measurement interval of 15 min (**a**–**c**) and the last 60 min (**d**–**f**). (**g**,**h**) Atomic-scale HAADF-STEM images of (**g**) PtNi, (**h**) PtCo and (**i**) Pt(NiCo) alloy NPs with good crystallinity and well-resolved lattice fringes confirming individual particles as perfect single crystals (insets are the assigned major facets, the calculated lattice spacings and FFT diffractograms).

Both PtNi and Pt(NiCo) NPs (Figure 1a,c,d,f and Supplementary Figure S1) exhibit four distinct morphologies (confirmed by assessments of populations of 400 NPs): 6-fold concave star-shaped (40% of PtNi and 36% of Pt(NiCo) NPs), 4-fold octahedral (35% of PtNi and 40% of Pt(NiCo)), 3-fold octapodal (10% of PtNi and 14% of Pt(NiCo)) and 6-fold tripodal (15% of PtNi and 10% of Pt(NiCo)) axes of symmetry. These structures were predominantly enclosed by {110} and {111} crystal planes, suggesting that crystal growth of the PtNi and Pt(NiCo) NPs occurred predominantly along <110> and <111> directions. This oriented growth further suggests that the {111} and {110} vertexes and edges served as foundations for selective adatom deposition [27,28]. In contrast, TEM analysis of PtCo NPs showed the formation of asymmetrically curved surface structures (Figure 1b,e and Supplementary Figure S1), suggesting restriction of the faceted morphologies seen in the PtNi and Pt(NiCo) NPs. We associate the PtCo NP morphology with the chemical reduction behaviour of the Co(Ac)_2_ precursor, with this large molecule preventing, due to steric hindrance, the formation of a faceted geometry [8]. The faceted morphology of Pt(NiCo) NPs, formed with the same Co precursor, suggests that PtNi nuclei formed rapidly and established a faceted morphology before Co incorporation during growth of the ternary NPs. The highly symmetric crystal growth of these PtNi and Pt(NiCo) NP populations may be attributed to preferential passivation/chemisorption of cosurfactants (OAm and HDA) on the least densely-packed {110} and most densely-packed {111} facets, contributing to unequal adatom growth rates/patterns on weakly passivated crystallographic planes. The use of binary surfactants thus allows growth rate differences between distinct crystal facets, producing mixtures of NP geometries with varying degrees of concavity. Atomic resolution high-angle annular dark field scanning transmission electron microscopy (HAADF-STEM) images (Figure 1g–i) of these alloy nanostructures reveal the crystalline lattice structures with a high degree of crystallinity, while continuous parallel and clearly-resolved lattice fringes indicate that the particles are perfect single crystals. The measured interplanar spacings (d) and the dominant facets are also indicated. Insets in Figure 1g–i are fast Fourier transforms (FFTs) which confirm that these alloy NPs are face centered cubic (FCC) single crystals despite their varying concavities. The bright regions covering the outer NP surface are associated with Pt-enrichment, while compositional inhomogeneity is evident across the individual alloy NPs.

## 6. Synthesis Using Mo(CO)_6_: The Effect of Time

We investigated the growth kinetics of these NPs by extracting colloidal aliquots during Mo(CO)_6_-assisted synthesis at uniform time intervals (15, 30, 45, and 60 min). Each of these samples was quenched immediately after extraction, to halt any structural changes, followed by the addition of absolute ethanol to separate the NPs from the synthesis medium. There was insignificant change in morphology or size as a function of synthesis time, confirming that particle growth was complete within the first 15 min of reaction time. Particle-size histograms (Supplementary Figure S2a–c) show narrow size distributions. The crystalline structures of the NPs, sampled at various reaction times, were investigated using PXRD as shown in Supplementary Figure S3a–c. The recorded PXRD peaks were assigned to the (111), (200), (220), (311), and (222) planes, consistent with (fcc) Pt. The slight positive shift of the peaks toward higher 2θ angles, relative to those of pure Pt, showed the formation of solid solutions of Pt with (smaller) Ni and/or Co atoms in the alloy NPs. These results are consistent with Vegard’s law [29,30]. There were no traces of impurities, segregated monometallic NPs, or asymmetric diffracting lines detected, demonstrating successful incorporation of solute metals into Pt crystal lattice. The diffraction peaks of these three alloy systems became sharper with extended ripening periods, suggesting improved NP crystallinity.

**Figure 2:**
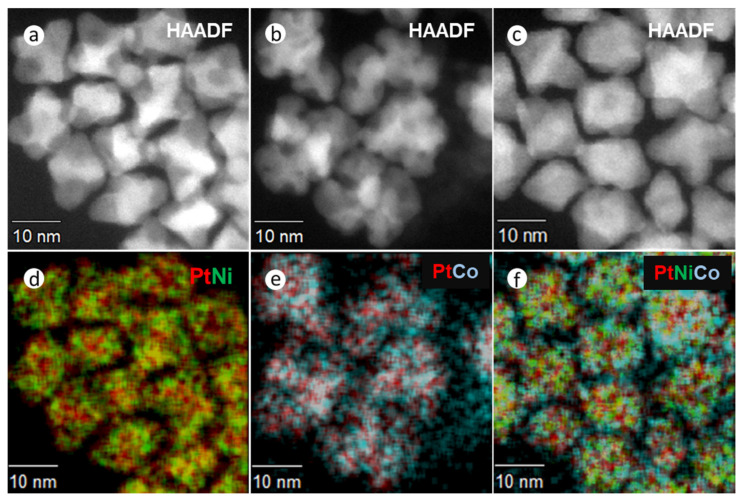
HAADF and STEM-EDS elemental distribution examination of PtNi, PtCo and Pt(NiCo) alloy NPs synthesized by Synthesis Method (a) (i). HAADF images of (**a**) PtNi, (**b**) PtCo and (**c**) Pt(NiCo) alloy NPs. (**d**–**f**) Composite EDX spectroscopic mappings of Ni (Kα, green), Co (Kα, cyan), and Pt (Lα, red) reveal inhomogeneous distributions of two solid solutions (Pt-rich and M-rich phases) within the alloy NPs.

To confirm the incorporation of solutes in these alloy NPs, elemental distribution was measured by STEM x-ray mapping (Figure 2 and Supplementary Figure S4). These figures show HAADF-STEM images and composite EDX spectroscopic mapping for PtNi (Figure 2a,d), PtCo (Figure 2b,e), and Pt(NiCo) (Figure 2c,f). All the NPs show compositional variations within the particles, i.e., an inhomogeneous solute-solvent distribution, with uneven bright regions (in the HAADF images) being characteristic of higher Pt concentrations, segregated to {111} surfaces. The darker regions were correspondingly enriched in both Ni and Co. These observations are consistent with the previous reports on inhomogeneous compositional variations [1,31,32]. The chemical composition of NPs was determined from STEM-EDS and the bulk elemental composition by ICP-OES; no consistent change in composition during the synthesis procedure was observed. Quantification (Supplementary Figure S4a–c) of the metal contents using the Cliff Lorimer ratio method and the K Factors supplied by the Noran software, showed mean elemental ratios of Pt_54_Ni_46_, Pt_48_Co_52_ and Pt_50_(Ni_26_Co_24_) (insets in EDS spectra, Supplementary Figure S4a–c). Supplementary Table S1 summarizes the particle sizes, crystallite domain sizes, and chemical compositions of these NPs at various synthesis times (15, 30, 45, 60 min). The average particle size of 250–300 PtNi, PtCo, and Pt(NiCo) NPs of extracted samples were measured from TEM micrographs of regions chosen at random and also from crystallite domain sizes estimated from PXRD data using the Debye–Scherrer equation.

## 7. Synthesis Using Mo(CO)_6_: The Effect of Precursor Salts

When the non-noble (solute) acetate metal precursors (Ni(ac)_2_ and Co(ac)_2_) used in (a) above, were replaced with the corresponding acetyl-acetonate (Ni(acac)_2_ and Co(acac)_2_) precursors, the synthesis resulted in monodisperse PtNi, PtCo and Pt(NiCo) NPs. TEM imaging shows that these NPs have octahedral morphologies (Supplementary Figure S5a–c). Unlike the situation when using of Ni and Co acetate salts, the simultaneous reduction of Pt(acac)_2_ in the presence of Ni(acac)_2_, Co(acac)_2_ or both, appeared to favour the consistent formation of octahedral morphologies bounded by {111} crystal facets. The average particle diameters of these NPs calculated from TEM images were 9.9 ± 1.1 nm (PtNi), 7.2 ± 0.5 nm (PtCo) and 6.7 ± 0.6 nm (Pt(NiCo)) (Supplementary Figure S5d–f). PXRD patterns of these three alloy NPs, showed fcc solid solution phases (Supplementary Figure S5g). Crystallite domain sizes estimated from PXRD data using the Debye-Scherrer equation were 8.2 nm (PtNi), 6.6 nm (PtCo), and 5.4 nm (Pt(NiCo)). These observations suggest that uniform-sized alloy NP systems can be achieved by a suitable choice of solute precursors in Mo(CO)_6_-assisted synthesis. We also note here that particle growth was complete within the first 15 min using these solute precursors, as we observed for Ni(ac)_2_ and/Co(ac)_2_ solute precursors.

## 8. Synthesis Using Mo(CO)_6_: The Effect of Surfactants

**Figure 3:**
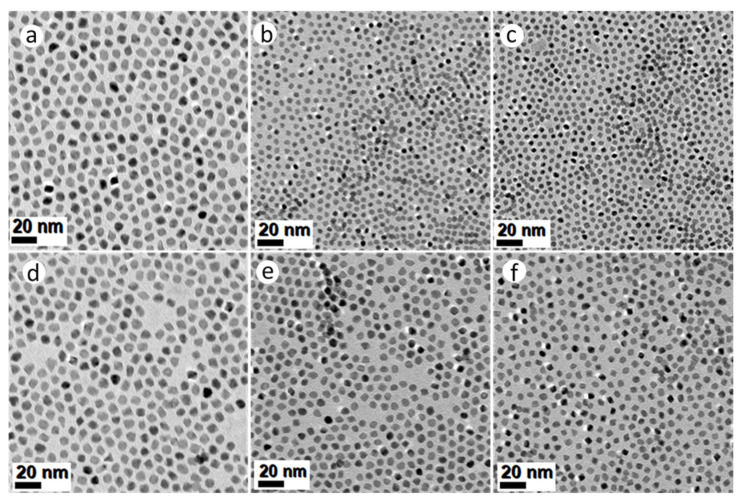
TEM BF images of PtNi, PtCo and Pt(NiCo) alloy NPs synthesized via simultaneous reduction of Pt(acac)_2_ and non-noble organometallic precursors: (**a**) Ni(Ac)_2_, (**b**) Co(Ac)_2_ and (**c**) Ni(Ac)_2_ + Co(Ac)_2_; (**d**) Ni(acac)_2_, (**e**) Co(acac)_2_ and (**f**) Ni(acac)_2_ + Co(acac)_2_ in the presence pure amine surfactant OAm using the reductant Mo(CO)_6_. All the corresponding PXRD lines of these PtNi, PtCo and Pt(NiCo) alloy NPs displayed single solid solution phases.

All the syntheses described thus far, have used a homogeneous mixture of surfactants oleylamine (OAm, 20 mL) and hexadecylamine (HDA, 4.4 g), in 25 mL BE. In the following experiments, this mixture was replaced with (a) pure OAm; and (b) 20 mL OAm mixed with 8.8 g HDA in 25 mL BE. The Mo(CO)_6_-assisted reduction of Pt(acac)_2_ in the presence of solute Ni(Ac)_2_ and/or Co(Ac)_2_ precursors in pure OAm, resulted in PtNi, PtCo and Pt(NiCo) NPs displaying well-defined octahedral geometries, as shown in Figure 3a–c. The average particle sizes calculated from TEM images were 8.8 ± 0.8 nm (PtNi), 4.5 ± 0.7 nm (PtCo) and 5.1 ± 0.6 nm (Pt(NiCo)) (Supplementary Figure S6a–c); whereas the crystallite sizes estimated from PXRD data (Supplementary Figure S6d) using the Scherrer equation were 7.3 nm (PtNi), 4.9 nm (PtCo) and 5.2 nm (Pt(NiCo)). The Mo(CO)_6_-assisted reduction of Pt(acac)_2_ in the presence of solute Ni(acac)_2_, Co(acac)_2_ and/or both precursors, using pure OAm resulted in NPs of similar octahedral shapes, as shown in Figure 3d–f.

The average sizes of these OAm-grown PtNi, PtCo and Pt(NiCo) alloy NPs were 9.6 ± 1.0 nm, 4.7 ± 0.6 nm and 6.4 ± 0.7 nm respectively (Supplementary Figure S7a–c). The crystallite domain sizes estimated from PXRD data (Supplementary Figure S7d) were 7.6 nm (PtNi), 4.5 nm (PtCo) and 5.9 nm (Pt(NiCo)). These findings suggest that the use of OAm as the colloidal stabilizer favours the formation of uniform-sized and thermodynamically favoured octahedral alloy NPs. Furthermore, pure OAm is known to selectively bind strongly on the {111} crystal facets, thus lowering the surface free energy of NPs and inducing the formation of energetically favoured octahedral geometries [21]. Further experiments were conducted in an attempt to increase the yields of high-index concave tripodal, octapodal, and star-shaped structural geometries, which have previously been reported to display outstanding electrocatalytic activities [33,34]. This was achieved by doubling the concentration of HDA. However, this resulted in tripodal yields of ~10–15% for both PtNi and Pt(NiCo) systems with no traces of concave star-shaped and octapodal surfaces: the majority of the NPs displayed octahedral morphologies. These findings suggest that HDA served an insignificant influence on the evolution of branched NP morphologies, instead it decreased the morphological yields and sizes of such nanostructures. On the other hand, the PtCo NPs exhibited close to 100% octahedral shape selectivity, unlike the irregular NPs observed for lower HDA concentration (Supplementary Figure S8a–c). These observations suggest that doubling the concentration of HDA forms smaller uniform-sized alloy NPs, thus slow growth rate of alloy NPs provided by HDA surface capping fostered thermodynamically favoured octahedral morphologies. The mean diameters of PtNi, PtCo and Pt(NiCo) alloy NPs were 10.4 nm (σ = 1.1), 5.4 nm (σ = 0.6) and 6.0 nm (σ = 0.8) respectively (Supplementary Figure S8d–f), indicating narrow NP size distributions. The PXRD patterns of these alloy types revealed fcc solid solution phases (Supplementary Figure S8g), with estimated crystallite domain sizes of: PtNi (7.3 nm, σ = 0.4), PtCo (5.5 nm, σ = 0.4), and Pt(NiCo) (5.8 nm, σ = 0.5).

## 9. Thermolytic Synthesis

**Figure 4:**
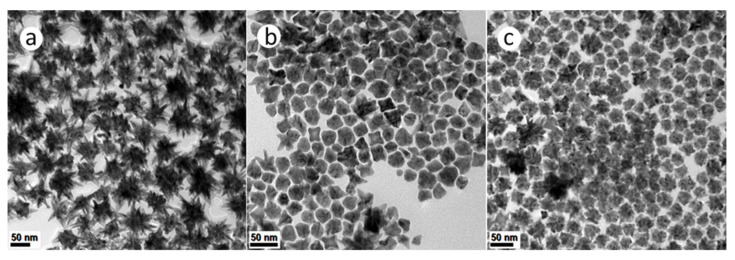
TEM BF images of PtNi, PtCo and Pt(NiCo) alloy NPs solution-grown via thermolytic decomposition of Pt(acac)_2_ in the presence of (**a**) Ni(Ac)_2_, (**b**) Co(Ac)_2_ and (**c**) Ni(Ac)_2_ + Co(Ac)_2_ in dual amine surfactants OAm and HDA.

High temperature thermolytic decomposition (i.e., without the use of reductants), was carried out using synthesis method (b). In this solution-phase synthetic approach, both temperature and the surfactants served to induce the reduction of metal precursors. Synthesis Method (b)(i) resulted in PtNi NPs with complex, hyperbranched structures as shown in Figure 4a. Both PtCo and Pt(NiCo) NPs (Figure 4b,c) showed the coexistence of asymmetrically-shaped (~90% Pt(NiCo) and ~95% PtCo) and dendritic (~10% Pt(NiCo) and ~5% PtCo NPs. The recorded PXRD patterns of these alloy types revealed fcc solid solution phases (Supplementary Figure S9).

## 10. Thermolysis: The Effect of Time

**Figure 5:**
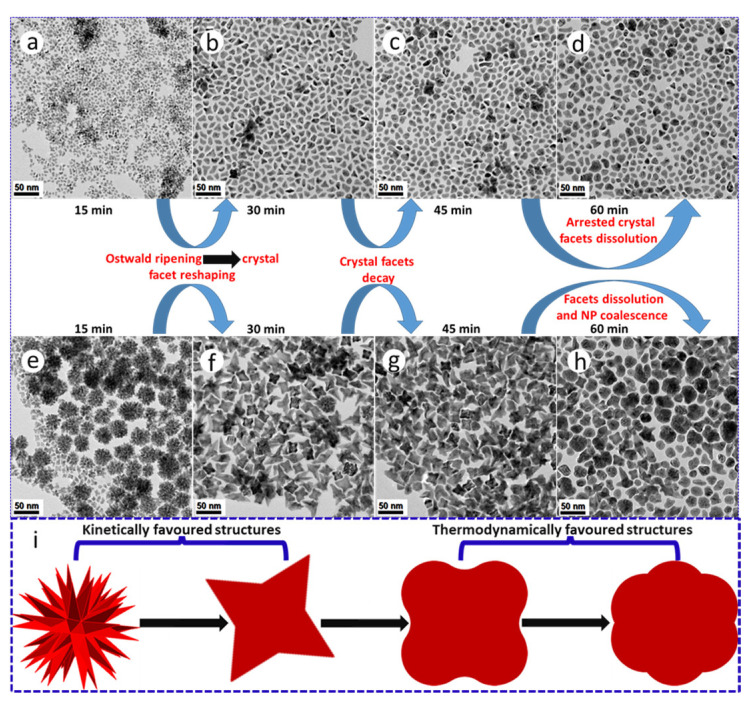
TEM BF images demonstrating the morphological transformations/changes of (**a**–**d**) PtNi and (**e**–**h**) Pt(NiCo) alloy NPs prepared by co-thermolytic decomposition of Pt and M (Ni and Co) acetylacetonate precursors. The colloidal alloy NP aliquots extracted from the bulk organic growth-mixture were sampled at different time intervals (from 15–60 min). (**i**) A corresponding schematic of geometrical restructuring from kinetically (high energy surface) to thermodynamically favourable (low energy surface) morphologies with extended ripening periods.

Our synthesis reaction times were initially shortened to 15 min as black products rapidly precipitated out of the bulk organic reaction medium, suggesting the formation of dense NPs and thus intrinsic colloidal instability. Based on the observation of high surface area PtNi and Pt(NiCo) polymorphs, we extended the reaction time to investigate the possibility of geometric reshaping or Ostwald ripening phenomena of these two-alloy systems. The ripening duration was extended to 60 min with aliquot samples extracted from the synthesis media after every 15 min. We observed dramatic morphological transformations from dendritic to near spherical surface structures with extended aging periods (Figure 5a–h). These reconstructive transformations/changes are attributed to minimization of NP total interfacial free energy [28,35,36,37]. TEM analysis reveals the evolution of dendritic structures in both systems, with the Pt(NiCo) alloy yielding more well-defined, high surface-area hyperbranched morphologies bounded by low surface energy {111} facets within the first 15 min of reaction (Figure 5g). However, both the PtNi and Pt(NiCo) systems show significant NP reshaping after 30 min (Figure 5b,f). The PtNi NPs evolved into dense irregular or near-spherical, convex and concave (monopods, bipods to tripods) shapes, with complete smoothing/dissolution of dendritic structures. After 45–60 min, the sample contained mostly spherical and random irregular shapes (Figure 5c,g). The Pt(NiCo) system displayed significant structural changes transitioning to intermediate geometries of cubes with concave surfaces on the major {100} crystal facets. These morphologies showed insignificant changes after a dwell time of 45 min. At 60 min, these alloy NPs morphed to near-spherical polydisperse morphologies (Figure 5d,h). These time-dependent ripening processes reveal that the NP surface reconstructions/transformations could arise due to the instability of the high-energy facets, particularly the rounding of the vertexes [38]. Furthermore, weakened/destabilized binding strength of colloidal stabilizers (surfactants) on the NPs at this dwell temperature (240 °C) may have permitted their morphological reshaping. In all cases, the development of dense and compact near-spherical NPs arises from the disappearance of high surface-area hyperbranched structures while most of the diverse anisotropic geometries evolved from the smaller colloids. In our studies, the results garnered from both PtNi and Pt(NiCo) NP systems clearly demonstrate that prolonged ripening periods promoted substantial geometric or reconstructive transformations of alloy colloids from kinetically (high energy surface) to thermodynamically favourable (low energy surface) morphologies [37,38], as shown in Figure 5i.

## 11. Thermolysis: The Effect of Precursor Salts

Under the same thermolytic conditions, the metal sources Ni(Ac)_2_ and Co(Ac)_2_ were replaced with Ni(acac)_2_ and Co(acac)_2_. TEM images showed the coexistence of alloy NP morphologies ranging from spherical, monopods, bipods, tripods, tetrapods to high surface-area dendritic structures (Figure 5a,b). Previous studies have shown that various single-crystalline and multi-crystalline structures with varying degrees of branch formation can coexist within the same synthesis media [37,39]. These diverse branched structures were observed to be more dominant in both PtNi and Pt(NiCo) NP populations (Figure 5b) than the asymmetrical morphologies of PtCo NPs with corroded surfaces (Supplementary Figure S10a). We observed a light blue solution during destabilization-purification of PtCo alloys using dry ethanol (as an antisolvent), suggesting the dissolution of cobalt (II) oxide (CoO). The formation of structures with multiple voids through the nanoscale Kirkendall-type effect [2,40,41,42,43], due to oxidized Co and its subsequent disintegration from the bulk PtCo system during the NP purification process, is an unusual phenomenon. Although TEM analysis revealed diverse NP morphologies, PXRD lines showed the crystalline nature of these three alloy types to be fcc solid solution phases. The PtCo diffraction peaks shifted towards lower 2θ values as a result of substantially dissolved Co/Co oxides (Supplementary Figure S10b). Stripping the surface-coordinating agents (stabilizers) from the crystal surfaces might have promoted the oxidation of Co atoms, consequently making them prone to disintegration from the PtCo nanostructure.

## 12. Thermolysis: The Effect of Surfactants

**Figure 6:**
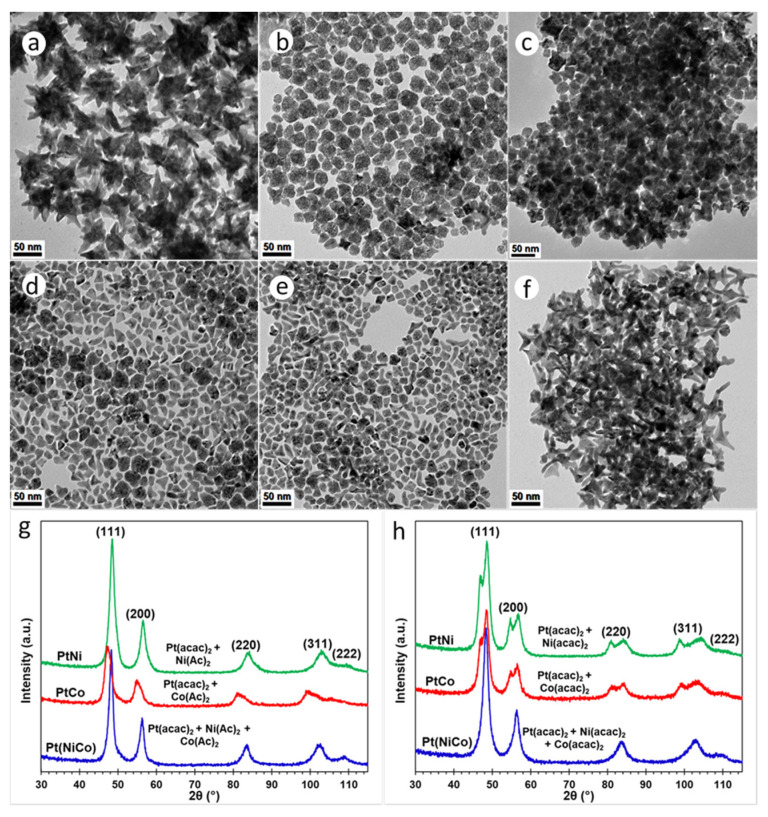
TEM BF images of PtNi, PtCo and Pt(NiCo) alloy NPs solution-grown via thermolytic decomposition of Pt(acac)_2_ with (**a**) Ni(Ac)_2_, (**b**) Co(Ac)_2_ and (**c**) Ni(Ac)_2_ + Co(Ac)_2_; (**d**) Ni(acac)_2_, (**e**) Co(acac)_2_ and (**f**) Ni(acac)_2_ + Co(acac)_2_ in amine OAm as the sole surfactant. (**g**) are the corresponding PXRD patterns of these PtNi, PtCo and Pt(NiCo) alloy NPs displayed single solid solution phases. (**h**) are PXRD patterns of Pt(NiCo) NPs showing diffraction lines of a single disordered solid solution whereas both PtNi and PtCo NP systems displayed splitting of the diffraction lines, demonstrating the co-existence of two compositionally-distinct fcc phases.

We investigated the effect of pure OAm on the crystal growth of alloy NPs under thermolytic decomposition conditions. The simultaneous thermolytic reduction of Pt(acac)_2_ in the presence of Ni(Ac)_2_ and Co(Ac)_2_ or both, resulted in the formation of various crystal facets. TEM analysis showed that the PtNi system produced dense dendritic NPs (Figure 6a). Both PtCo and Pt(NiCo) NPs displayed misshapen (50% PtCo and 40% Pt(NiCo)) and mostly thermodynamically stable near-spherical (50% PtCo and 60% Pt(NiCo)) geometries (Figure 6b,c). These observations suggest that more energetically favourable surface structures evolved and thus, restricted kinetic controlled growth modes of alloy NPs. The branching growth observed in the PtNi alloys is restricted for both PtCo and Pt(NiCo) systems, possibly due to steric hindrance or differences in the chemical reduction kinetics associated with Co(Ac)_2_ precursor used [8]. When organometallic precursors (Ni(acac)_2_ and Co(acac)_2_) were used during synthesis, the PtNi NPs exhibited dendritic morphologies. Both PtCo and Pt(NiCo) NPs produced kinked monopods and tripods; the majority of the NP populations exhibited mostly inhomogeneous (random) crystal facets (Figure 6d–f). These observations suggest that the morphological evolution of branched NPs is inhibited for the PtCo and Pt(NiCo) alloys. Similarly, we correlate this phenomenon with the steric effects provided by the use of precursor Co(acac)_2_ [8]. PXRD patterns of Pt(NiCo) and PtNi NPs show single solid solutions, whereas the PtCo NP population displays asymmetric diffraction peaks, with the Pt-rich phase at lower 2θ values diffracting more sharply than the Co-rich phase. We again observed the evolution of a light blue solution during PtCo alloy purification process, suggesting the dissolution of Co/Co oxides from the PtCo alloy. TEM analysis revealed porous PtCo NPs because of Kirkendall effects induced through substantial dissolution of Co (II) oxide (Figure 6b) and hence the characteristic PtCo diffraction peak shift towards lower 2θ values (Figure 6g).

PXRD analyses of these NP populations synthesized by co-decomposing Pt(acac)_2_ and solute (Co(Ac)_2_, Ni(Ac)_2_ or both) precursors showed that the Pt(NiCo) system displayed symmetric diffraction lines of a single disordered solid solution whereas both PtNi and PtCo systems display splitting of the diffraction lines, demonstrating the existence of two compositionally-distinct fcc phases (Figure 6h). These split diffraction lines of PtNi and PtCo NPs show that the diffracting Pt-rich segregated phases (at lower 2θ values) coexist with intensely sharp diffracting lines for Ni- or Co-rich phases (at higher 2θ values). The preferential elemental segregation of Pt to the NP vertices and surfaces can be expected as Pt has a larger lattice parameter (0.393 nm) than Ni (0.352 nm) [44] or Co (0.355 nm) so this segregation will reduce the total lattice strain of the system. The estimated atomic compositions of PtNi NPs from the (111) plane using Vegard’s rule [29,30] (assuming *d*_Pt_ = 0.393 nm and *d*_Ni_ = 0.352 nm) at 2θ split diffraction angles of 47.1° and 48.7° contain Pt_85_Ni_15_ and Pt_50_Ni_50_ alloy phases, respectively. Furthermore, the (111) plane of PtCo NPs (assuming *d*_Co_ = 0.355 nm) at 2θ split diffraction lines of 47.1° and 48.6° show Pt-rich (Pt_84_Co_16_) and Co-rich (Pt_47_Co_53_) phases, respectively. It is worth noting that the symmetric and diffracting line of Pt(NiCo) alloys is in accordance with the sharp diffracting Ni- or Co-rich phases, demonstrating ~Pt_50_(NiCo)_50_ elemental composition.

These findings indicate that the onset of morphological evolution under these thermolytic reaction environments occurred under kinetic growth regime coupled with Ostwald ripening. Prolonged ripening periods promoted the transformation of high surface area or energy facets (edges, kinks, and corners) into thermodynamically stable alloy geometric configurations with reduced total interfacial free energies. Thus, the surface stress induced on these high-energy facets deformed the initial morphologies of the alloys to more stable near-round geometries, manifesting in spontaneous minimization of the total surface free energy due to surface reconstruction or termination of the high-energy surface facets. We also observe the retention of some metastable-branched structures with varying degrees of curvature within the same synthesis reactions, indicating incomplete reconstructive transformation over these extended dwelling times and temperatures. Accordingly, this thermolytic approach poses synthesis challenges associated with the manipulation of uniform crystal structures (sizes and shapes), demanding rigorous tuning of the reduction kinetics to achieve a balanced homogeneous crystal nucleation and subsequent NP growth regime.

## 13. Conclusions

We developed a facile one-pot Mo(CO)_6_-assisted synthetic approach for the development of binary and ternary Pt-based alloy NPs. The use of cosurfactants (OAm and HDA) allowed the evolution of a variety of NP morphologies with varying degrees of curvature. These were predominantly concave star-shaped, octahedral, octapodal, and tripodal morphologies in PtNi and Pt(NiCo), whereas the PtCo system yielded irregularly-shaped particles. Preferential binding or surfactant exchange of OAm and HDA thus allows growth rate differences between distinct crystal facets. Variations in solute metal precursors and exclusive use of OAm produced alloy NPs of octahedral {111}-dominated crystal facets. For all the synthesis variables reported here, the average edge-lengths of PtNi NPs are larger than those of PtCo and Pt(NiCo) NPs. We attribute these size differences to the steric effects or distinct reduction kinetics provided by the Co (acetate and acetylacetonate) precursors used during NP synthesis. Our findings demonstrate that this Mo(CO)_6_-assisted co-reduction of different metal cations enabled the preparation of substitutional solid solution NPs with tunable sizes and well-defined geometries for potential applications in heterogeneous catalysis.

## Supplementary Materials

The following are available online at https://www.mdpi.com/article/10.3390/nano11071825/s1: Figure S1. STEM BF images of PtNi, PtCo and Pt(NiCo) alloy NPs. Figure S2. Particle edge-length distributions of PtNi, PtCo and Pt(NiCo) alloy NPs at various sampling intervals (15, 30, 45 and 60 min). Figure S3. The PXRD patterns of PtNi, PtCo and Pt(NiCo) alloy NPs extracted from the colloidal growth mixture at various time intervals (15, 30, 45 and 60 min). Figure S4. HAADF images (grey) of PtNi, PtNi and Pt(NiCo) NPs: elemental maps for Ni (Kα, green) map, Co (Kα, blue) map and Pt (Lα, red) map. EDS spectra and the insert is quantified elemental compositions. Figure S5. TEM BF images, particle edge-length distributions and the corresponding PXRD patterns of these PtNi, PtCo and Pt(NiCo) alloy NPs. Figure S6. Particle edge-length distributions and the corresponding PXRD patterns of PtNi, PtCo and Pt(NiCo) alloy NPs. Figure S7. Particle edge-length distributions and the corresponding PXRD patterns of PtNi, PtCo and Pt(NiCo) alloy NPs. Figure S8. TEM BF images, particle edge-length distributions and the corresponding PXRD patterns of PtNi, PtCo and Pt(NiCo) alloy NPs. Figure S9. PXRD patterns of PtNi, PtCo and Pt(NiCo) alloy NPs solution-grown via thermolytic decomposition. Figure S10. TEM BF image of surface-etched and agglomerated PtCo alloy NPs and PXRD patterns of PtNi, PtCo and Pt(NiCo) alloy NPs. Table S1. The particle size, crystallite domain size and chemical compositions of PtNi, PtCo and Pt(NiCo) alloy NPs.
