# Supplementary material for: Formation of Pt-Based Alloy Nanoparticles Assisted by Molybdenum Hexacarbonyl
Source: Nanomaterials (Basel). 2021 Jul 14;11(7):1825. doi: 10.3390/nano11071825 (PMC8308230; doi:10.3390/nano11071825)
Supplement: Supplementary file 1 [file nanomaterials-11-01825-s001.zip › nanomaterials-1267190-supplementary.pdf]

nanomaterials

MDPI

Article

Formation of Pt-Based Alloy Nanoparticles Assisted by Molybdenum Hexacarbonyl

Gerard M. Leteba [1,2], David R. G. Mitchell [3], Pieter B. J. Levecque [1], Eric van Steen [1] and Candace I. Lang [2,*]

1 Catalysis Institute, Department of Chemical Engineering, University of Cape Town, Cape Town 7700, South Africa; gerard.leteba@uct.ac.za (G.M.L.); Pieter.levecque@uct.ac.za (P.B.J.L.); eric.vansteen@uct.ac.za (E.v.S.)
2 School of Engineering, Macquarie University, Sydney, NSW 2109, Australia; candace.lang@mq.edu.au
3 Electron Microscopy Centre, Innovation Campus, University of Wollongong, Wollongong, NSW 2517, Australia; dmitchel@uow.edu.au
* Correspondence: gerard.leteba@uct.ac.za (G.M.L); candace.lang@mq.edu.au (C.I.L)

Citation: Leteba, G.M.; David R. G. Mitchell; Pieter B. J. Levecque; van Steen, E.; Lang, C.I. Formation of Pt-Based Alloy Nanoparticles Assisted by Molybdenum Hexacarbonyl. *Nanomaterials* 2021, *11*, 1825. https://doi.org/10.3390/nano11071825

Academic Editors: Eva Pellicer; Vincenzo Amendola; Diego Cazorla-Amorós

Received: 3 June 2021
Accepted: 7 July 2021
Published: 14 July 2021

Publisher's Note: MDPI stays neutral with regard to jurisdictional claims in published maps and institutional affiliations.

Copyright: © 2021 by the authors. Submitted for possible open access publication under the terms and conditions of the Creative Commons Attribution (CC BY) license (http://creativecommons.org/licenses/by/4.0/).

1. Experimental Methods

1.1. Chemicals and Materials.

The solution-phase synthetic approach involved the following metal precursors (all purchased from Sigma-Aldrich): platinum acetylacetonate ($Pt(acac)_2$, 97%), nickel (II) acetate tetrahydrate ($Ni(Ac)_2$, 98%), cobalt (II) acetate tetrahydrate ($Co(Ac)_2$, ≥99%), nickel (II) acetylacetonate ($Ni(acac)_2$, 95%) and cobalt (II) acetylacetonate ($Co(acac)_2$, 97%). Oleylamine (OAm, 70%) and hexadecylamine (HDA, 90%) were employed as surfactants and benzyl ether (BE, 98%), as a high-boiling point solvent. Solvents (Sigma-Aldrich) such as absolute ethanol, acetone and chloroform, used for precipitating, cleaning and re-dispersing the particles, were all of analytical grade. All the chemicals were used as-received without any further purification.

2. Thermal Decomposition of Organometallic Precursors.

(a) Synthesis of binary Pt-based nanoparticles.

In a typical synthesis: precursor salts $Pt(acac)_2$ (0.08g, 0.2 mmol) + $Ni(Ac)_2$ (0.048g, 0.2 mmol) and $Pt(acac)_2$ (0.08g, 0.2 mmol) + $Co(Ac)_2$ (0.048g, 0.2 mmol) were dissolved in OAm (20 ml) and HDA (4.4g) (surfactants) using BE (25 ml) as the high boiling point solvent. The resulting metal salt-surfactant-solvent reaction mixture was heated at 150 °C for 5–10 minutes under vigorous magnetic stirring in a round bottom flask. The resultant pale-yellow homogeneous organic solution turned dark brown during the heat-up process to 240 °C and thereafter black, with a heating rate of 10 °C/min. The colloidal mixture was held at this temperature for 60 min. Thereafter, the colloidal medium was removed from the heat source and quenched on ice. Subsequently, the as-synthesized NPs were extracted from the synthesis media through flocculation by adding excess absolute ethanol and chloroform. After settling (typically 1–2 days), the excess organic solvents were decanted and the particles were further cleaned by re-suspending in absolute ethanol. This colloidal refining process was performed 3 times. The black product was finally re-dispersed in chloroform, yielding a black colloidal suspension.

Under the same synthesis conditions, ternary Pt(NiCo) NPs were synthesized by dissolving $Pt(acac)_2$ (0.08g, 0.2 mmol) + $Ni(Ac)_2$ (0.024g, 0.1 mmol) + $Co(Ac)_2$ (0.024g, 0.1 mmol) in OAm (20 ml) + HDA (4.4g) + BE (25 ml). The simultaneous thermal reduction process is similar to the one described above for the preparation of binary alloy NPs.

(b) Synthesis of binary and ternary Pt-based nanoparticles.

In a typical synthesis: precursor salts $Pt(acac)_2$ (0.08g, 0.2 mmol) + $Ni(acac)_2$ (0.052g, 0.2 mmol), $Pt(acac)_2$ (0.08g, 0.2 mmol) + $Co(acac)_2$ (0.052g, 0.2 mmol) and $Pt(acac)_2$ (0.08g, 0.2 mmol) + $Ni(acac)_2$ (0.026g, 0.1 mmol) + $Co(acac)_2$ (0.026g, 0.1 mmol) were dissolved in

dual surfactants OAm (20 ml) and HDA (4.4g) in BE (25 ml). The resulting metal salt-surfactant-solvent reaction mixture was heated at 150 °C for 5–10 minutes under vigorous magnetic stirring in a round bottom flask. The resultant pale-yellow homogeneous solution turned dark brown to black during the heat-up process to 240 °C, with a heating rate of 10 °C/min. The resultant colloidal mixture was held at this temperature for 60 min.

(c) Synthesis of binary and ternary Pt-based nanoparticles.

In a typical synthesis: precursor salts $Pt(acac)_2$ (0.08g, 0.2 mmol) + $Ni(Ac)_2$ (0.048g, 0.2 mmol), $Pt(acac)_2$ (0.08g, 0.2 mmol) + $Co(Ac)_2$ (0.048g, 0.2 mmol) and/or $Pt(acac)_2$ (0.08g, 0.2 mmol) + $Ni(Ac)_2$ (0.024g, 0.1 mmol) + $Co(Ac)_2$ (0.024g, 0.1 mmol) were dissolved in pure OAm (20 ml) + BE (25 ml). The resulting metal salt-surfactant-solvent reaction mixture was heated at 150 °C for 5–10 minutes under vigorous magnetic stirring in a round bottom flask. The bulk organic synthesis mixture then turned dark brown to black during the heat-up process to 240 °C, with a heating rate of 10 °C/min. The resultant colloidal mixture was held at this temperature for 60 min.

Under the same synthesis conditions, both binary and ternary NPs were synthesized by dissolving $Pt(acac)_2$ (0.08g, 0.2 mmol) + $Ni(acac)_2$ (0.052g, 0.2 mmol), $Pt(acac)_2$ + $Co(acac)_2$ (0.052g, 0.2 mmol) and $Pt(acac)_2$ (0.08g, 0.2 mmol) + $Ni(acac)_2$ (0.026g, 0.1 mmol) + $Co(acac)_2$ (0.026g, 0.1 mmol) in pure amine OAm (20 ml) + BE (25 ml). The co-thermal reduction process is similar to the one described above.

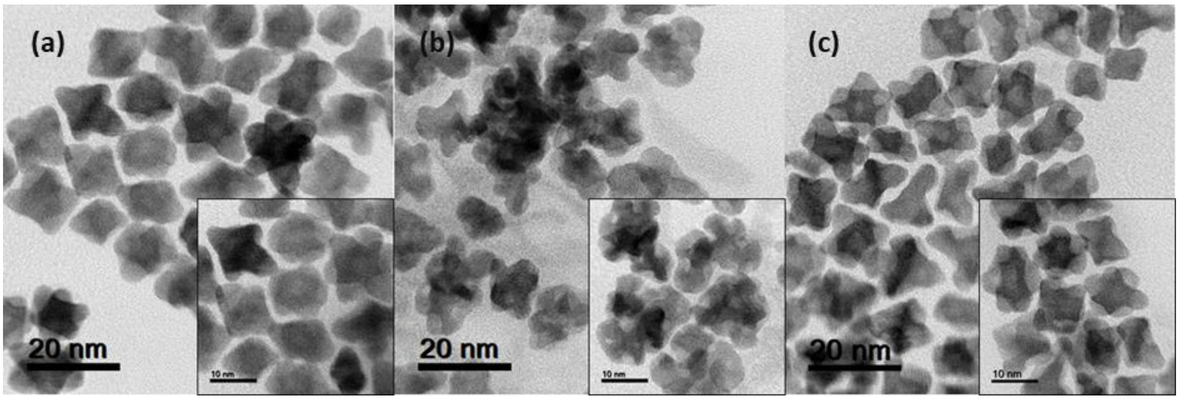

Figure S1. STEM BF images of (**a**) PtNi, (**b**) PtCo and (**c**) Pt(NiCo) alloy NPs obtained after 60 min of reaction time. The inserts are enlarged BF-STEM micrographs.

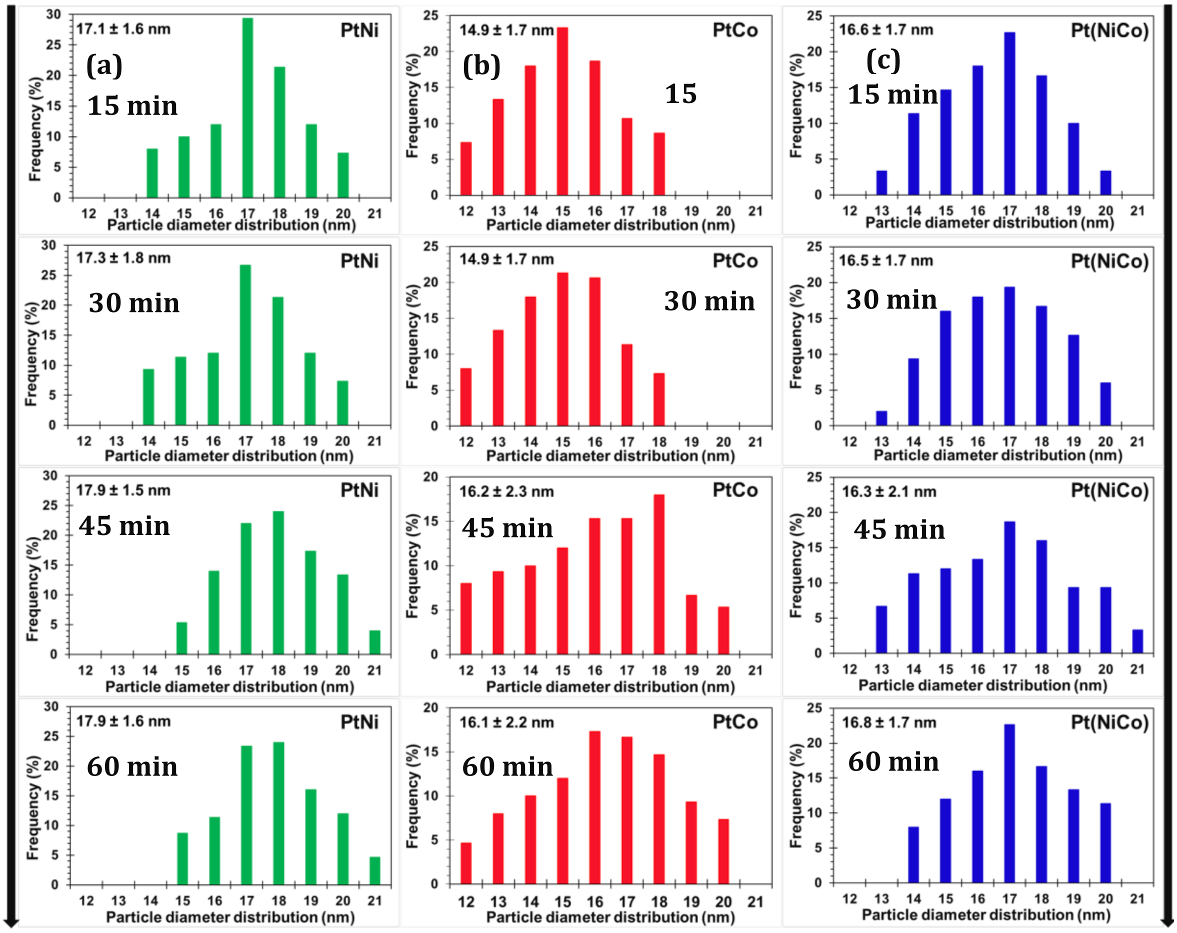

Figure S2. Particle edge-length distribution of these three types of alloys: (**a**) PtNi, (**b**) PtCo and (**c**) Pt(NiCo) NPs, solution-grown via simultaneous reduction of Pt(acac)$_2$ with (**a**) Ni(Ac)$_2$, (**b**) Co(Ac)$_2$ and (**c**) Ni(Ac)$_2$ + Co(Ac)$_2$ in the presence of cosurfactant OAm using Mo(CO)$_6$ as the reductant. The alloy nanoparticles were sampled by extracting aliquots from the bulk organic growth-mixture of colloidal alloy NPs at various time intervals (15, 30, 45 and 60 min).

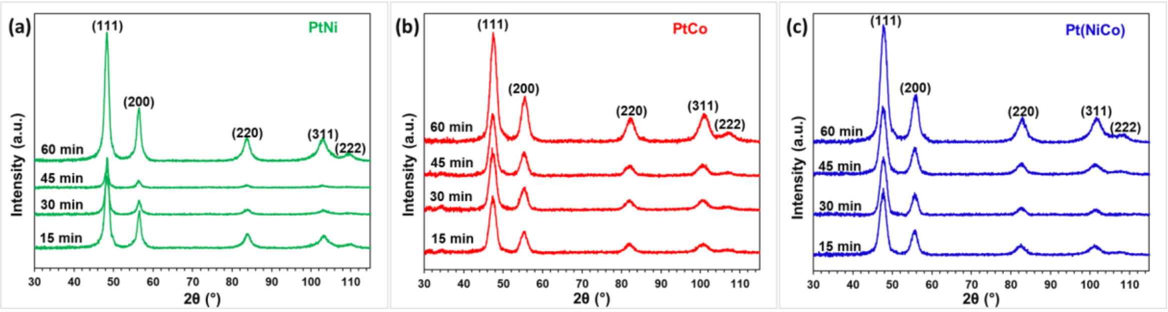

Figure S3. The PXRD patterns of (**a**) PtNi, (**b**) PtCo and (**c**) Pt(NiCo) alloy NPs extracted from the colloidal growth mixture at various time intervals (15, 30, 45 and 60 min).

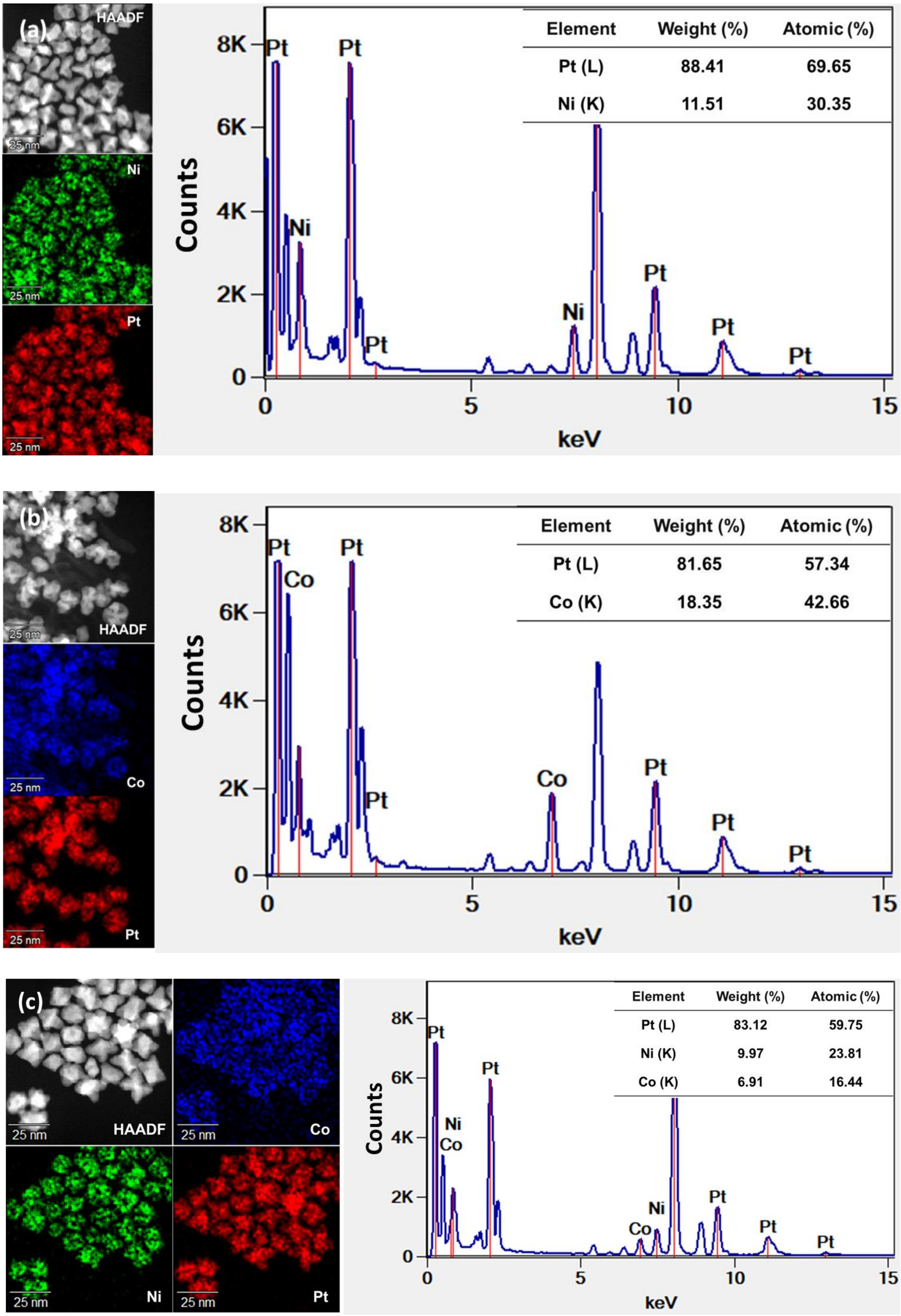

Figure S4. HAADF images (grey) of (**a**) PtNi, (**b**) PtNi and (**c**) Pt(NiCo) NPs: elemental maps for Ni (Kα, green) map, Co (Kα, blue) map and Pt (Lα, red) map. EDS spectra and the insert is quantified elemental compositions.

Table S1. The effect of synthesis time on the particle size, crystallite domain size and chemical compositions of PtNi, PtCo and Pt(NiCo) alloy NPs.

Reaction Time (min)	Particle Size (nm), TEM			Crystallite Size (nm), XRD		
	Pt(NiCo)	PtCo	PtNi	Pt(NiCo)	PtCo	PtNi
15	16.3 ± 1.7	14.9 ± 1.7	17.1 ± 1.6	15.2	14.5	17.3
30	16.5 ± 1.7	14.9 ± 1.7	17.3 ± 1.8	14.7	14.9	17.9
45	16.3 ± 2.1	16.2 ± 2.3	17.9 ± 1.5	14.5	14.7	17.5
60	16.8 ± 1.7	16.1 ± 2.2	17.9 ± 1.6	15.5	15.3	18.2
**Reaction time (min)**	**Composition (at.%), STEM-EDS**			**Composition (at.%), ICP-OES**		
	**Pt(L):Ni(K):Co (K)**	**Pt(L):Co(K)**	**Pt(L):Ni(K)**	**Pt:Ni:Co**	**Pt:Co**	**Pt:Ni**
15	65:22:13	53:47	55:45	60:30:10	51:49	56:44
30	64:22:14	59:41	54:46	61:26:13	53:47	51:49
45	51:20:29	58:42	49:51	54:25:21	54:46	52:48
60	50:26:24	48:52	54:46	56:24:20	49:51	48:52

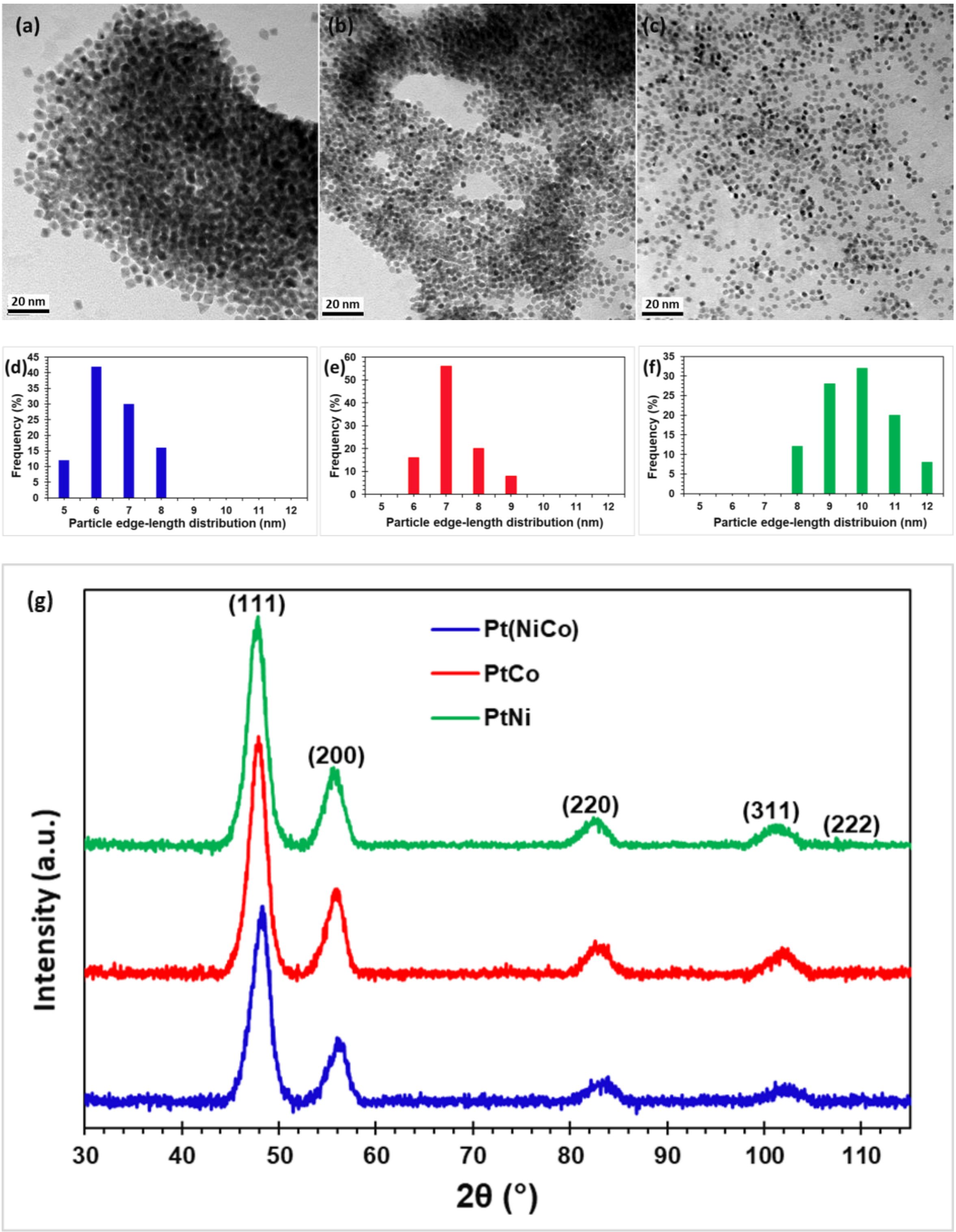

Figure S5. TEM BF images of (**a**) PtNi, (**b**) PtCo and (**c**) Pt(NiCo) alloy NPs synthesized via simultaneous reduction of $Pt(acac)_2$ in the presence of $Ni(acac)_2$, $Co(acac)_2$ and $Ni(acac)_2 + Co(acac)_2$ in amine surfactants (OAm + HDA) and using the reductant $Mo(CO)_6$. Particle edge-length distribution of these three types of alloys: (**d**) PtNi, (**e**) PtCo and (**f**) Pt(NiCo) NPs. (**g**) All the corresponding PXRD patterns of these PtNi, PtCo and Pt(NiCo) alloy NPs showed single solid solution phases.

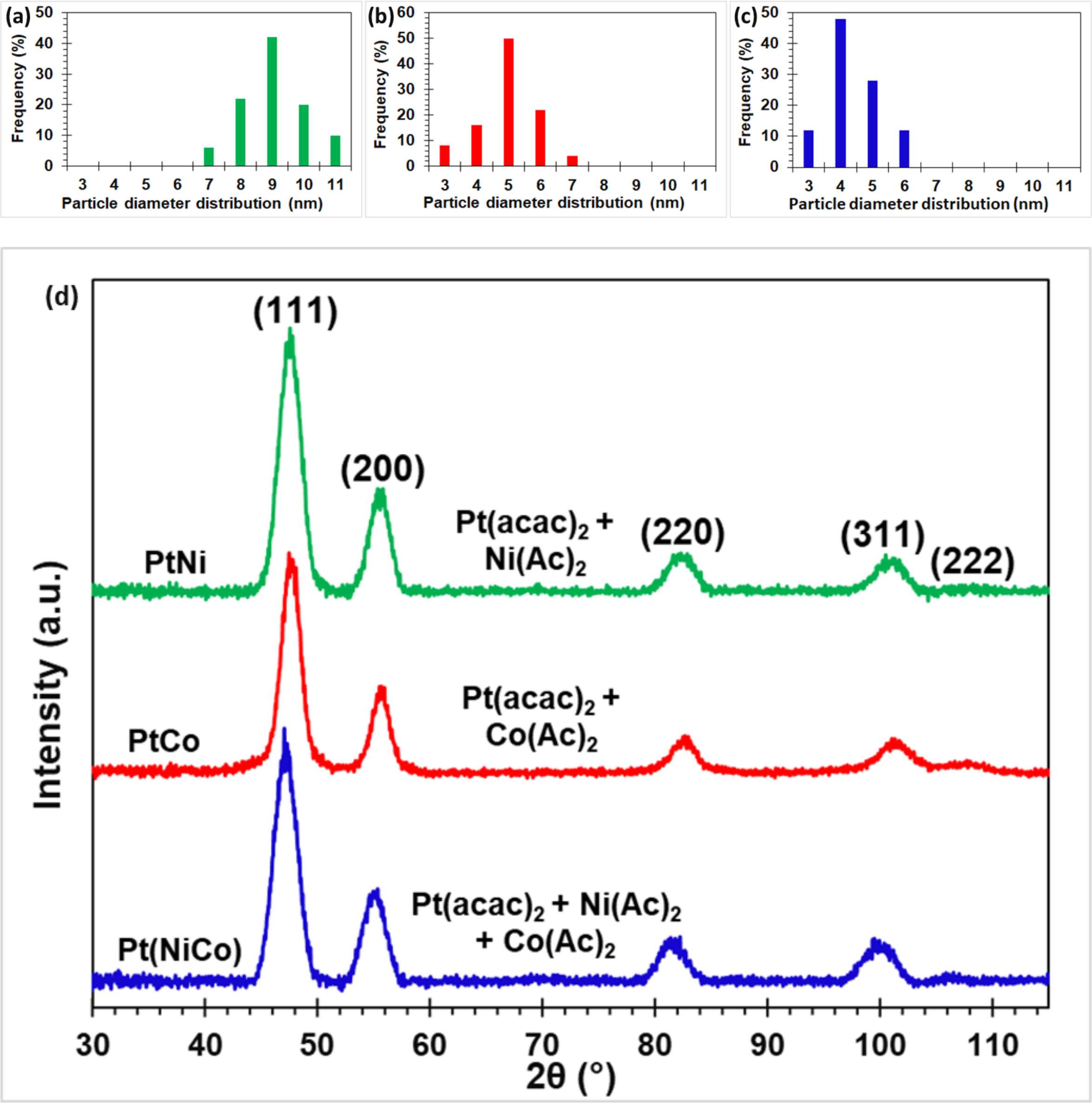

Figure S6. Particle edge-length distribution of these three types of alloys: (**a**) PtNi, (**b**) PtCo and (**c**) Pt(NiCo) NPs, solution-grown via simultaneous reduction of Pt(acac)$_2$ in the presence of Ni(Ac)$_2$, Co(Ac)$_2$ and Ni(Ac)$_2$ + Co(Ac)$_2$ in the presence of pure amine surfactant OAm using Mo(CO)$_6$ as the reductant. (**d**) PXRD patterns of these PtNi, PtCo and Pt(NiCo) alloy NPs showed single solid solution phases.

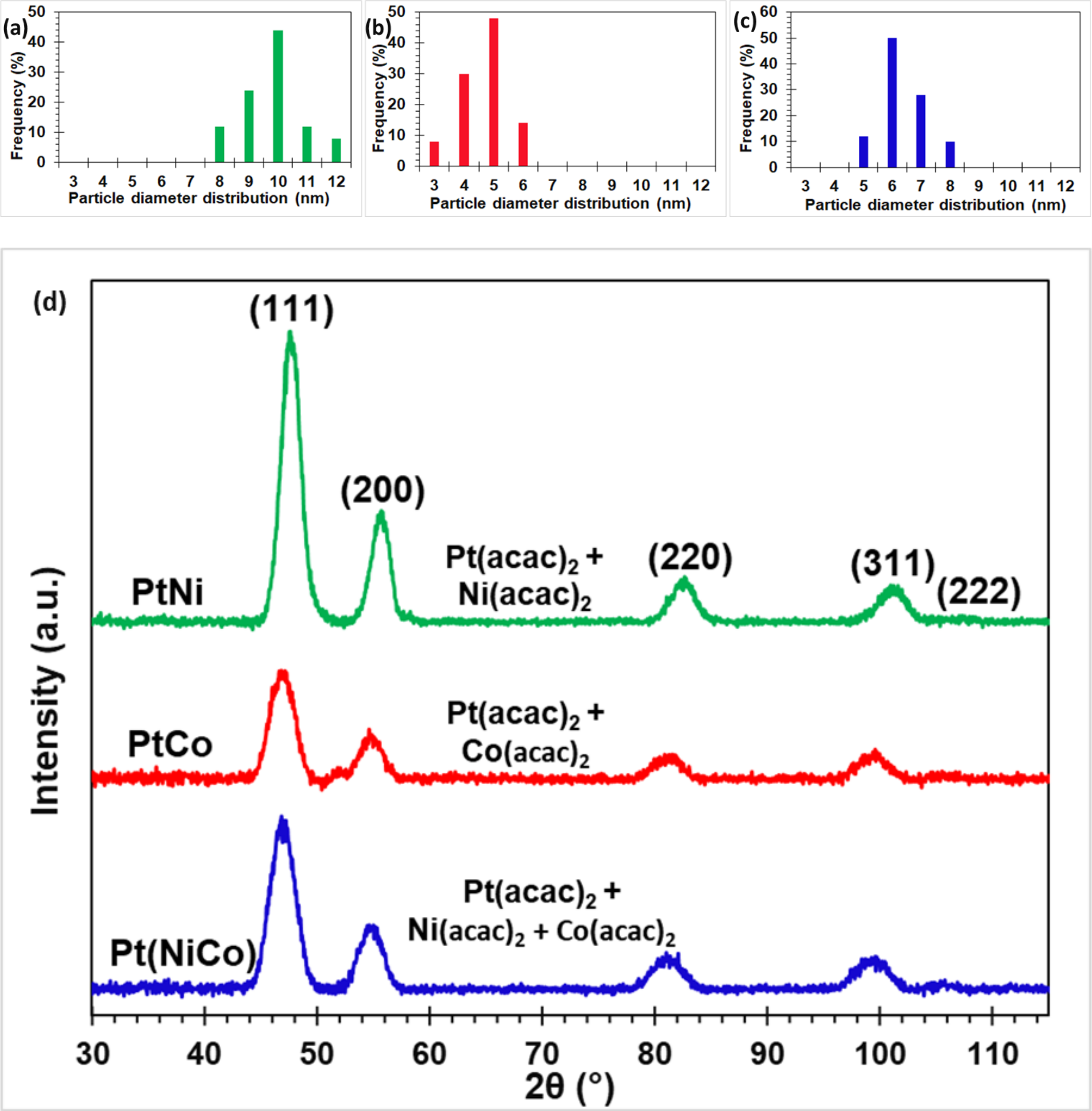

Figure S7. Particle edge-length distribution of these three types of alloys: (**a**) PtNi, (**b**) PtCo and (**c**) Pt(NiCo) NPs, solution-grown via simultaneous reduction of Pt(acac)$_2$ with Ni(acac)$_2$, Co(acac)$_2$ and Ni(acac)$_2$ + Co(acac)$_2$ in the presence of pure amine surfactant OAm with Mo(CO)$_6$ serving as Table S8. TEM BF images of (**a**) PtNi, (**b**) PtCo and (**c**) Pt(NiCo) alloy NPs synthesized via simultaneous reduction of Pt(acac)$_2$ with non-noble organometallic precursors: Ni(Ac)$_2$, Co(Ac)$_2$ and Ni(Ac)$_2$ + Co(Ac)$_2$ in the presence amine surfactants OAm and doubled HDA concentration using the reductant Mo(CO)$_6$. Particle edge-length distribution of these three types of alloys: (**d**) PtNi, (**e**) PtCo and (**f**) Pt(NiCo) NPs. (**g**) All the corresponding PXRD patterns of these PtNi, PtCo and Pt(NiCo) alloy NPs displayed single solid solution phases.

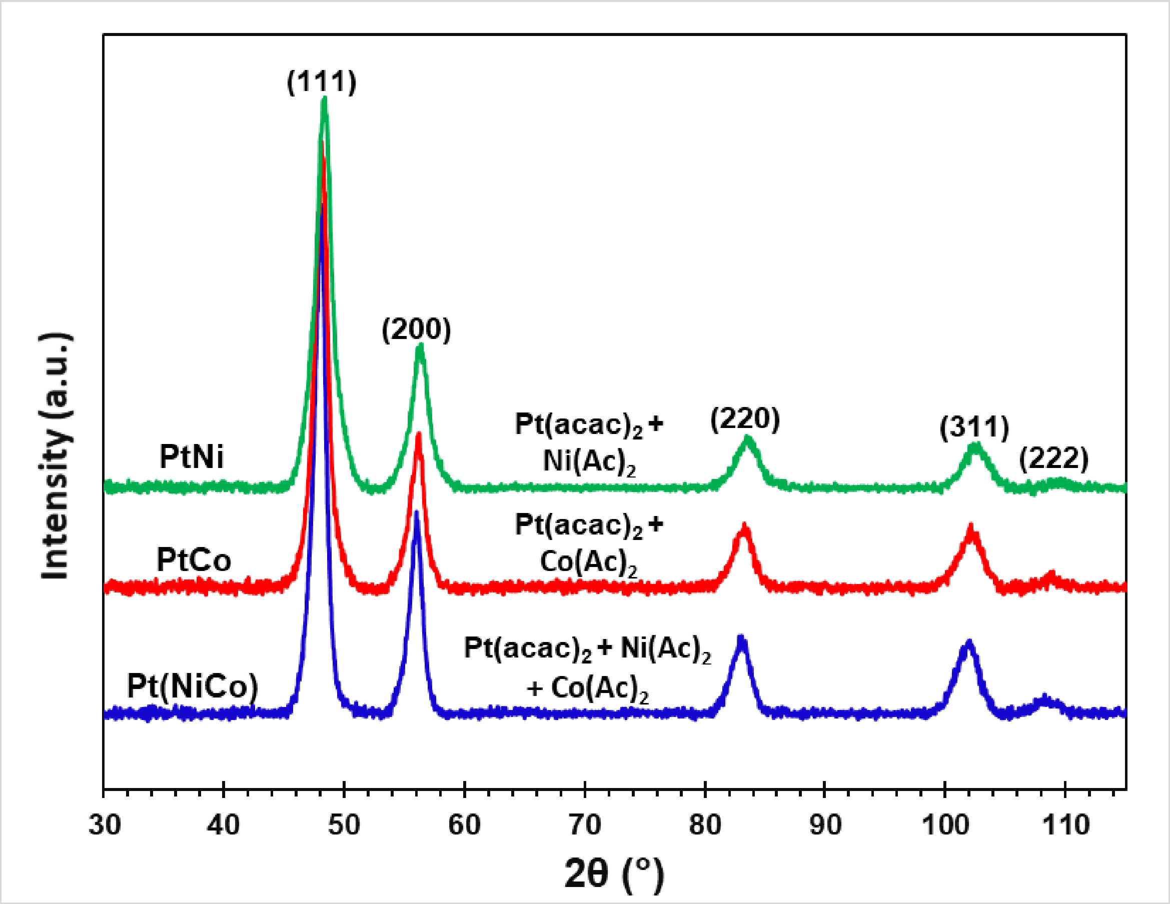

Figure S9. PXRD patterns of PtNi, PtCo and Pt(NiCo) alloy NPs solution-grown via thermolytic decomposition of Pt(acac)$_2$ in the presence of Ni(Ac)$_2$, Co(Ac)$_2$ and Ni(Ac)$_2$+ Co(Ac)$_2$ in dual amine surfactants OAm and HDA. All the PXRD patterns of these alloy NPs showed single solid solution phases.

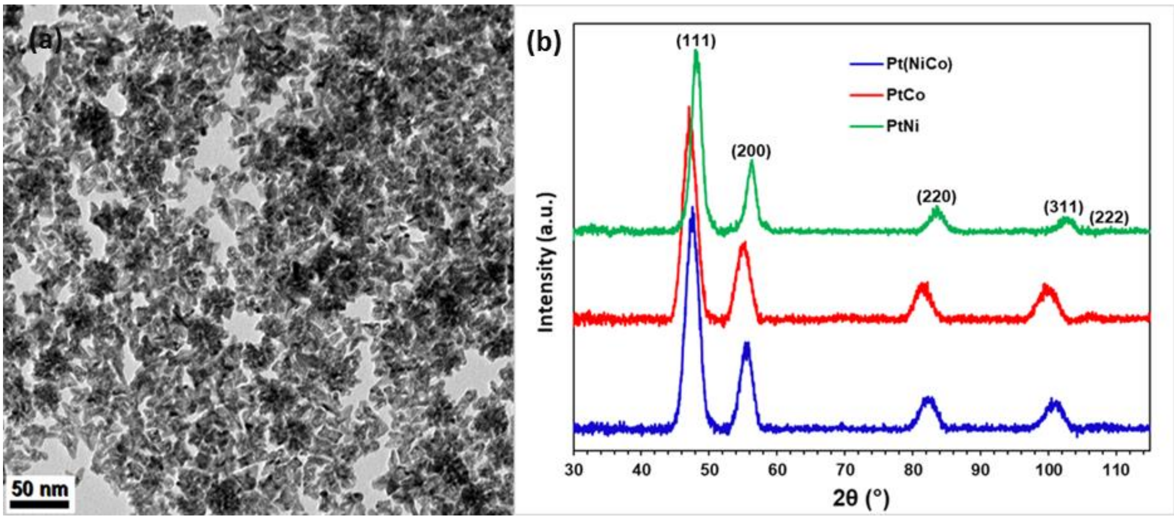

Figure S10. (**a**) A typical TEM BF image of surface-etched and agglomerated PtCo alloy NPs synthesized via thermolytic decomposition of Pt(acac)$_2$ and Co(acac)$_2$ in dual amine surfactants (OAm and HDA). (**b**) All the corresponding PXRD patterns of these PtNi, PtCo and Pt(NiCo) alloy NPs displayed single solid solution phases.

3. XRD Analysis Using Vegard's Law and Bragg's Law:

3.1. Vegard's Law

The expression for Vegard's law[1,2] for a binary solid solution A–B is:

$$d_{alloy} = Xd_A + (1 - X)Xd_B \quad (1)$$

where $X = XB$ is the mole fraction of component B, Xd_A and $Xd_B$are the lattice constants of pure components A and B, respectively.

3.2. Calculations Based on the XRD Diffraction Peak Positions

3.2.1. PtNi:

(1) Lattice constant = 0.386968 nm at 2θ split diffraction peaks of 47.1°:

$$\text{Pt: } 0.3869 = 0.393(X) + (1 - X)0.352 \quad (1)$$

$$X = 85\ at.\% \quad (2)$$

$$\text{Ni: } 0.3869 = 0.352(X) + (1 - X)0.393 \quad (1)$$

$$X = 15\ at.\% \quad (2)$$

(2) Lattice constant = 0.3725 nm at 2θ split diffraction peaks of 48.6°:

$$\text{Pt: } 0.373 = 0.393(X) + (1 - X)0.352 \quad (1)$$

$$X = 50\ at.\% \quad (2)$$

$$\text{Ni: } 0.373 = 0.352(X) + (1 - X)0.393 \quad (1)$$

$$X = 50\ at.\% \quad (2)$$

3.2.2. PtCo:

(1) Lattice constant = 0.3872 nm at 2θ split diffraction peaks of 47.1°:

$$\text{Pt: } 0.387 = 0.393(X) + (1 - X)0.355 \quad (1)$$

$$X = 84\ at.\% \quad (2)$$

$$\text{Co: } 0.387 = 0.352(X) + (1 - X)0.393 \quad (1)$$

$$X = 16\ at.\% \quad (2)$$

(2) 2Lattice constant = 0.3725 nm at 2θ split diffraction peaks of 48.7°:

$$\text{Pt: } 0.373 = 0.393(X) + (1 - X)0.355 \quad (1)$$

$$X = 47\ at.\% \quad (2)$$

$$\text{Co: } 0.373 = 0.352(X) + (1 - X)0.393 \quad (1)$$

$$X = 53\ at.\% \quad (2)$$

References

1. Vegard, L. Die Konstitution der Mischkristalle und die Raumfüllung der Atome. *Z. Phys.* **1921**, *5*, 17–26, doi:10.1007/BF01349680.
2. Denton, A.R.; Ashcroft, N.W. Vegard's law. *Phys. Rev. A* **1991**, *43*, 3161–3164, doi:10.1103/PhysRevA.43.3161.
